# Data Aggregation Based on Overlapping Rate of Sensing Area in Wireless Sensor Networks

**DOI:** 10.3390/s17071527

**Published:** 2017-06-29

**Authors:** Xiaolan Tang, Hua Xie, Wenlong Chen, Jianwei Niu, Shuhang Wang

**Affiliations:** 1 College of Information Engineering, Capital Normal University, Beijing 100048, China; tangxl@cnu.edu.cn (X.T.); 2151002064@cnu.edu.cn (H.X.); 2 State Key Laboratory of Virtual Reality Technology and Systems, School of Computer Science and Engineering, Beihang University, Beijing 100191, China; niujianwei@buaa.edu.cn; 3 Research Institute of Beihang University in Shenzhen, Shenzhen 518057, China; 4 Schepens Eye Research Institute, Harvard Medical School, Boston, MA 02114, USA; shuhang_wang@meei.harvard.edu

**Keywords:** wireless sensor networks, data aggregation, overlapping rate of sensing area, data accuracy

## Abstract

Wireless sensor networks are required in smart applications to provide accurate control, where the high density of sensors brings in a large quantity of redundant data. In order to reduce the waste of limited network resources, data aggregation is utilized to avoid redundancy forwarding. However, most of aggregation schemes reduce information accuracy and prolong end-to-end delay when eliminating transmission overhead. In this paper, we propose a data aggregation scheme based on overlapping rate of sensing area, namely AggOR, aiming for energy-efficient data collection in wireless sensor networks with high information accuracy. According to aggregation rules, gathering nodes are selected from candidate parent nodes and appropriate neighbor nodes considering a preset threshold of overlapping rate of sensing area. Therefore, the collected data in a gathering area are highly correlated, and a large amount of redundant data could be cleaned. Meanwhile, AggOR keeps the original entropy by only deleting the duplicated data. Experiment results show that compared with others, AggOR has a high data accuracy and a short end-to-end delay with a similar network lifetime.

## 1. Introduction

Wireless sensor networks (WSNs) consist of a large quantity of sensor nodes to offer a variety of services, such as environmental monitoring and security surveillance [1,2]. Nowadays, WSNs are considered as one of the most promising technologies for cyber manufacturing systems in Industrial Internet of Things (IIoT) [3]. In smart factories, WSNs serve for intelligent industrial control applications in harsh environments [4,5]. In order to provide highly reliable and realtime transmission, sensor nodes are often densely distributed in monitoring areas. However, the high density of node deployment causes lots of redundant data, and hence their forwarding brings in a large waste of the limited power and bandwidth, resulting in low energy efficiency and short network lifetime.

In order to avoid the transmissions of redundant information, data aggregation is required in WSNs. In most of aggregation schemes, the whole network is separated into several areas like grids according to the geographical coordinates, and then the data collected by sensors in each area are aggregated by a particular node [6]. Because of the possible random distribution of nodes as well as the fixed size and shape of the aggregation area, the similarity of data collected by different sensors in one area is not close, which affects the performance of aggregation. Additionally, since there may be multiple hops from an ordinary node to an aggregation node, the redundant data might be forwarded for several hops and hence lead to a high energy cost. Besides, an aggregation node has to wait for collecting all sensor data in its area before aggregation, which results in a long end-to-end delay [7,8].

In this paper, we propose a novel data aggregation scheme based on overlapping rate of sensing area in WSNs, named AggOR, to achieve energy-efficient data collection and keep high data accuracy. With respect to the sensing ranges, several nodes construct a gathering area if their overlapping rates of sensing area are no less than a preset threshold. In this way, the data in a gathering area have relatively high correlation and hence would be aggregated efficiently to eliminate redundancy. Moreover, there are only one or two levels of nodes in a gathering area. Usually a lower-level node transfers its sensing data to an upper-level node which aggregates and then delivers data to the sink. Therefore, redundant data are removed immediately after one-hop forwarding, which prohibits more energy consumption of redundancy relay. For further energy saving, an appropriate neighbor node at the same level could also be selected as the aggregation node if subject to particular conditions in the aggregation rules.

The main advantages of our scheme are listed below. (1) Construct gathering areas according to the overlapping rate of sensing area. It helps to remove a large quantity of duplicated data and keep almost all entropy of the original information; (2) Three aggregation rules take full advantage of data aggregation by selecting aggregating nodes from candidate parent nodes or appropriate neighbor nodes. They limit the hops of redundancy forwarding and decrease the transmission overhead as much as possible; (3) A large quantity of experiments show that AggOR scheme keeps a high accuracy of data, improves energy efficiency and achieves a quick data collection.

The rest of this paper is organized as follows. In Section 2, some related work on data aggregation in WSNs is discussed. Definitions and aggregation rules used in AggOR are introduced in Section 3, and Section 4 details the implementation of AggOR. Experimental results are analyzed in Section 5, and Section 6 concludes this paper.

## 2. Related Work

Data aggregation is utilized in WSNs to diminish the resource consumption of redundant data when delivering information from sensor nodes to the sink. In specific, data aggregation schemes could be classified into three kinds, i.e., tree-based aggregation [9,10,11,12], hybrid aggregation [13], and cluster-based aggregation [14,15,16,17,18,19,20,21,22].

In classical tree-based data aggregation schemes, a spanning tree rooted at the sink is constructed firstly, and then data are forwarded from leaves to root along the paths in the tree. In Tiny AGgregation (TAG) [10], after leaf nodes send their own data to their parent, the parent node aggregates data from its children and delivers the aggregated data to the root. Obviously, TAG is inefficient in case of dynamic topologies or link/device failures. In [11], Deligiannakis et al. propose an aggregation tree construction/reorganization algorithm to minimize energy cost. By calculating and sending a small set of intuitive statistics, a parent node may be substituted by one of its sibling nodes based on attachment cost. In [12], an adaptive spanning tree algorithm (AST) is proposed, which adaptively builds and adjusts an aggregation spanning tree. Owing to the strategies of random waiting times and alternative father nodes, AST establishes a relatively balanced spanning tree with flexible adjustments. Considering that a single packet, as the output of aggregation algorithm at a given level of the tree, may stand for all the data coming from a subtree, if it is lost, the entropy from this subtree might be lost as well.

As a typical hybrid data aggregation scheme, Tributary-Delta [13] combines the advantages of tree and multi-path by implementing them simultaneously in different regions of the network. It supports region adjustment in response to network condition changes, and determines the number of useful aggregates in the scenario. However, it may have a high overhead because of frequent update of the data gathering structure.

Compared with tree-based and hybrid schemes, cluster-based aggregation schemes usually have good scalability and high energy efficiency [14,15]. Considering that the cluster heads, which are close to the sink, relay data for others, in [16], Li et al. propose an energy-efficient unequal clustering scheme (EEUC). Cluster heads are elected by localized competition, and the competition range becomes small when it is near the base station. Therefore, those clusters closer to the sink have smaller sizes than others, and the energy consumption of cluster heads is balanced. Even though, the cluster maintenance is somewhat difficult.

In recent years, some cluster-based schemes analyze various factors to select a cluster head from several candidates. In DHCR [17], energy consumption, adjustment degree and exact distance from sensors to the base station are three main parameters for cluster head selection. Multi-hop routing and clustering are combined to decrease the number of control packets. In [18], Leu et al. propose REAC-IN to evenly distribute cluster heads based on the residual energy of each sensor and the average energy of sensors in the cluster. Together with isolated node checking considering power and distance, REAC-IN improves the cluster head selection process and avoids node isolation. To save energy for cluster reformation, Yi and Yang propose Hamilton energy-efficient routing protocol (HEER) [19]. Members in each cluster are linked on a Hamilton Path, and take turns to work as cluster head. In this way, no cluster reformation is required. However, the clusters are formed like LEACH in the first round, which may cause energy hole problem; the condition that all members in a cluster could communicate with each other is strict; the intra-cluster nodes in the Hamilton Path transmitting data in turn prolong the end-to-end delay.

Moreover, some existing studies exploit spatial correlation to set aggregation areas. In YEAST algorithm, those nodes detecting the same event are grouped in a cluster and the cluster head is the node closest to the sink [20]. The cluster is divided into spatially correlated cells, and only one node within each cell transfers its data to its cluster head, which stand for all the sensing data in this cell. Cells can be resized dynamically according to the application requirements. However, the bigger cells become, the less the entropy is. Since a representative node’s sensing area could not cover the whole cell, YEAST causes low accuracy to some extent. For several synchronous events, DRINA [21] tends to maximize the number of aggregation nodes and decrease the overhead of control packets. Nodes sensing the same event form a cluster, and a route for a new event is connected with an already established route which has the shortest path between them. Experiment results show that DRINA has a high aggregation rate, and reliable data aggregation and transmission. Nevertheless, it increases the overload of nodes in existing routes, and thus leads to unbalanced energy consumption and even the energy-hole problem.

In the above aggregation schemes, those nodes located in a particular area usually compose a cluster. If the area is too large, the similarity of data gathered by member nodes is small; if the area is too small, the advantage of data aggregation is degraded. In order to balance aggregation efficiency and data accuracy, we explore the relation of sensing areas of nodes to deal with redundant data, and utilize a threshold of overlapping sensing area to guarantee high accuracy after data aggregation.

## 3. Network Model and Aggregation Rules

### 3.1. Network Model

In this paper, we assume that all sensor nodes have the same sensing radius, denoted by $R_{S}$, and the same communication radius, denoted by $R_{C}$ ($R_{C}>2R_{S}$) [23]. The data collected by sensor networks may be periodic sensing information, such as the average temperature, or information triggered by specific events, such as fire alerts. Our scheme focuses on the periodic data collection, and assumes that the amount of information collected is the same for all the nodes, which is a common assumption about data collection [24]. The data collected by each node is denoted by *d*. Since the sink usually has sufficient power, we only consider the energy cost of sensor nodes in our scheme. Because data sending (the amount of data sent out from node $v_{i}$ is denoted by $d_{i}^{T}$) and receiving (the amount of data received by node $v_{i}$ is denoted by $d_{i}^{R}$) consume most of the energy, small energy consumptions such as the cost of data processing are ignored. Therefore we focus on the transmission overhead in AggOR. For simplicity, we assume that total energy consumption of node $v_{i}$ is $E_{i}^{C}=d_{i}^{T}E_{T}+d_{i}^{R}E_{R}$, where $E_{T}$ and $E_{R}$ are the energy costs for sending and receiving per unit data, respectively. The primary symbols used in AggOR are listed in Table 1.

Before presenting the details of AggOR, several definitions are introduced as follows.

**Definition** **1.** *Transmission hierarchy diagram: The diagram is a directed acyclic graph, including all the sensors, the possible communication paths, and the hierarchy levels (denoted by L). It is similar to a tree structure rooted at the sink, but the parent node is not unique.* 

**Figure 1:**
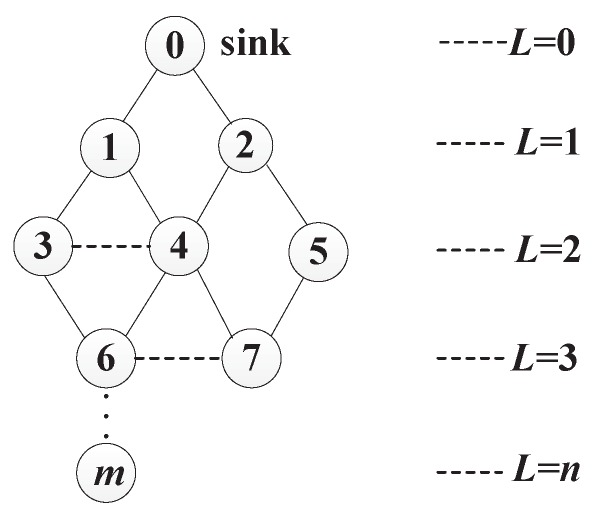
Transmission hierarchy diagram.

For data collection in a dense network, we only care about those nodes which can communicate with the sink through one-hop or multi-hop transmissions. For two levels $L_{i}$ and $L_{i}+1$ in the diagram, $L_{i}$ is called upper-level and $L_{i}+1$ is called lower-level. In other words, the value of upper-level is smaller than that of lower-level. As shown in Figure 1, there are *m* sensor nodes in the network as well as $v_{0}$ as the sink. The edges show the communication chances between nodes. Specifically, the solid lines indicate the possible parent-child relations between lower-level and upper-level nodes, while dotted lines show the neighbor relations between nodes at the same level.

**Definition** **2.** *Overlapping rate of sensing area, $OR_{i,j}$: The ratio of the overlapping sensing area of two nodes $v_{i}$ and $v_{j}$ to a node’s entire sensing range ($\pi {R_{S}}^{2}$).*

Furthermore, considering that the two nodes with a larger overlapped sensing area probably have a larger similarity of the collected data, we assume that the amount of duplicated data at two sensors is proportional to the overlapping rate of sensing area. Our aggregation only removes redundant data, and thus the entropy of the sensing data in the whole network is not lost. In other words, the aggregation works like a lossless compression approach whose compression ratio is the overlapping rate of sensing area.

In order to construct gathering areas, a threshold of overlapping rate of sensing area, denoted by $OR_{T}$, is utilized. The assignment of $OR_{T}$ affects the aggregation efficiency. The smaller $OR_{T}$ is, the larger the gathering area is, but the smaller the amount of duplicated data between nodes is. Further analysis of $OR_{T}$ is in Section 5.3.2.

**Figure 2:**
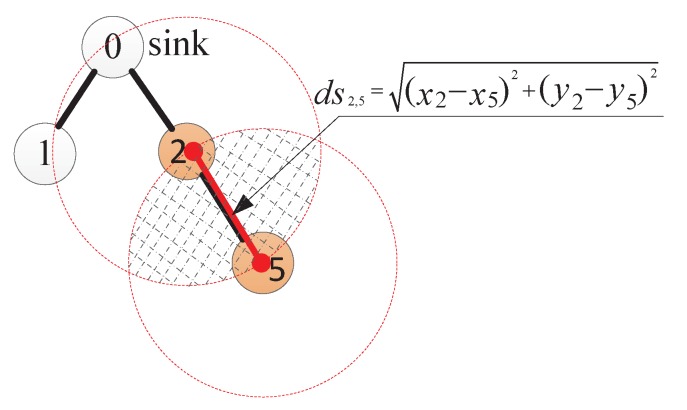
Overlapping rate of sensing area.

As shown in Figure 2, take the overlapping sensing area of $v_{2}$ and $v_{5}$ in Figure 1 as an example. Two dashed circles represent the sensing ranges of two nodes, respectively, and the hatched area is the overlapping sensing area, denoted by $S_{C}$. Therefore the overlapping rate of sensing area of $v_{2}$ and $v_{5}$ is computed by $OR_{2,5}=\frac{S_{C}}{\pi {R_{S}}^{2}}$. In order to calculate $S_{C}$, we denote the distance between $v_{i}$ and $v_{j}$ by $ds_{i,j}$. According to geometric theory, the overlapping sensing area is $S_{C}=2{R_{S}}^{2}\;arccos\frac{ds_{i,j}}{2R_{S}}-\frac{ds_{i,j}}{2}\sqrt{4{R_{S}}^{2}-{ds_{i,j}}^{2}}$. To remove the complicated calculation of inverse trigonometric functions, using curve fitting mechanism [25], $S_{C}$ could be computed as $S_{C}=0.34{ds_{i,j}}^{2}-2.26R_{S}ds_{i,j}+\pi {R_{S}}^{2}$. Therefore, $OR_{i,j}=\frac{0.34{ds_{i,j}}^{2}}{\pi {R_{S}}^{2}}-\frac{2.26ds_{i,j}}{\pi R_{S}}+1$.

**Definition** **3.** *Gathering area, GA: A gathering area is composed of a gathering node and several member nodes, and the overlapping rate of sensing area between the gathering node and each member node equals or is larger than $OR_{T}$.* 

The gathering node is responsible for collecting and aggregating all the sensor data in the gathering area and then sending the result toward the sink, while the member node transfers its data to the gathering node. A gathering area with node $v_{i}$ as its gathering node and $v_{j}$,…, $v_{k}$ as its member nodes is expressed by $GA_{i}=\left(v_{i},\left\{v_{j},\ldots ,v_{k}\right\}\right)$. One node belongs to at most one gathering area. If a node $v_{k}$ does not find a gathering node, $v_{k}$ turns into an independent node and forms a gathering area by itself as $GA_{k}=\left(v_{k},\emptyset \right)$. If a node is not a gathering node, it is called non-gathering node; if a node does not join in a gathering area, it is called free node.

**Definition** **4.** *Candidate parent nodes of $v_{i}$ , $CP_{i}$: A set consists of the nodes at level $L_{i}-1$ and in the communication range of $v_{i}$.* 

**Definition** **5.** *Neighbor nodes of $v_{i}$, $N_{i}$: A set includes the nodes at level $L_{i}$ which can communicate with $v_{i}$.* 

**Definition** **6.** *Candidate gathering nodes of $v_{i}$, $CG_{i}$: A set includes all the nodes which might be the gathering node of $v_{i}$. In AggOR, $CG_{i}\subseteq CP_{i}\cup N_{i}$.* 

**Figure 3:**
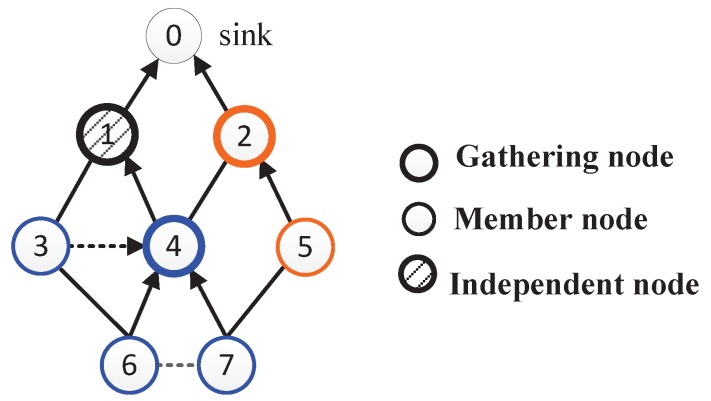
An instance of wireless sensor network (WSN) with three gathering areas.

As an instance, Figure 3 shows a transmission hierarchy diagram having three gathering areas, i.e., $GA_{4}=\left(v_{4},\left\{v_{3},v_{6},v_{7}\right\}\right)$, $GA_{2}=\left(v_{2},\left\{v_{5}\right\}\right)$, and $GA_{1}=\left(v_{1},\emptyset \right)$. The arrows indicate the directions of data transfers. Therefore, $v_{3}$, $v_{6}$ and $v_{7}$ send their data to $v_{4}$ which aggregates these data with its own data, and then sends the results to the sink; $v_{5}$ transfers its data to $v_{2}$, and $v_{2}$ aggregates these data; $v_{1}$ sends its data to the sink directly.

### 3.2. Aggregation Rules

We have three aggregation rules for the gathering area construction. Although in general, $E_{R}$ is slightly smaller than $E_{T}$ which is relevant with the transmission distance, to simplify the explanations of these rules, we assume $E_{R}=E_{T}=E$ and E·d=e.

**Rule 1.** *In gathering areas, the upper-level nodes have priorities over the lower-level nodes to be selected as gathering nodes.*

We evaluate the validity of Rule 1 in an instance gathering area with a upper-level node $v_{j}$ and a lower-level node $v_{i}$. Apparently, $L_{i}=L_{j}+1\geq 2$.

Case 1: $v_{i}$ is the gathering node. After $v_{j}$ joins the gathering area $GA_{i}$, $v_{j}$ as the member node sends *d* data to $v_{i}$. Thus $d_{j}^{R}=0$, and $d_{j}^{T}=d_{i}^{R}=d$. After data aggregation, $v_{i}$ transmits additional $\gamma \cdot d(0<\gamma =1-OR_{i,j}<1)$ data as well as its own data *d*. Hence, $d_{i}^{T}=\left(1+\gamma \right)d$. Since these data need to be forwarded to the sink through $L_{i}$ hops. The energy consumed for delivering aggregated data from the gathering node $v_{i}$ is $E_{GN_{i}}=\left(2L_{i}-1\right)d_{i}^{T}\cdot E=\left(2L_{i}-1\right)\left(1+\gamma \right)e$. Therefore, the total energy consumption of those nodes in the gathering area $GA_{i}$ is computed by

(1) $$\begin{array}{cc} E_{GA_{i}} & =d_{j}^{T}\cdot E+d_{i}^{R}\cdot E+E_{GN_{i}}\\ & =[\left(2L_{i}-1\right)\left(1+\gamma \right)+2]e. \end{array}$$

Case 2: $v_{j}$ is the gathering node. Similarly, the total energy cost of data delivery from those nodes in $GA_{j}$ to the sink is

(2) $$\begin{array}{cc} E_{GA_{j}} & =[\left(2L_{j}-1\right)\left(1+\gamma \right)+2]e\\ & =[\left(2L_{i}-3\right)\left(1+\gamma \right)+2]e. \end{array}$$

Obviously $E_{GA_{j}}<E_{GA_{i}}$. Because only replicated data are cleaned in aggregation, no matter which node is the gathering node, the quantities of information after aggregation are the same. As a result of a higher level of the gathering node, Case 1 has more energy consumed than Case 2. Thus selecting the gathering nodes from the upper-level nodes is better than from the lower-level nodes.

**Rule 2.** *After the lower-level nodes complete the construction of gathering areas, the free nodes at the upper level firstly select their gathering nodes from the candidate parent nodes. If no suitable candidate parent node exists, then select from the neighbor nodes.*

Assume that $v_{k}$ (the current node) is selecting a gathering node. $v_{j}$ is its candidate parent node, while $v_{i}$ is a neighbor node of $v_{k}$. Hence $L_{k}=L_{i}=L_{j}+1$.

Case 1: $OR_{i,k}\geq OR_{T}$ and $OR_{j,k}\geq OR_{T}$. In this case, $v_{k}$ select $v_{i}$ or $v_{j}$ to be its gathering node. If $v_{i}$ is the gathering node of $v_{k}$, $v_{i}$ receives data of size *d* from $v_{k}$ and transfers additional $\alpha \cdot d(0<\alpha =1-OR_{i,k}<1)$ data as well as its own data after aggregation. The total energy cost of transmitting data from $v_{k}$ to the sink via $v_{i}$ is

(3) $$E_{i}^{k}=\left[\left(2L_{k}-1\right)\alpha +2\right]e.$$

Otherwise, if $v_{k}$ chooses $v_{j}$ as its gathering node, the total energy cost for data delivery from $v_{k}$ to the sink via $v_{j}$ is

(4) $$E_{j}^{k}=\left[\left(2L_{k}-3\right)\beta +2\right]e,$$

where $0<\beta =1-OR_{j,k}<1$.

Comparing $E_{i}^{k}$ and $E_{j}^{k}$, we know that if α≥β (in other words, $OR_{i,k}\leq OR_{j,k}$), then $E_{i}^{k}>E_{j}^{k}$, and $E_{i}^{k}$ is larger than $E_{j}^{k}$ by at least 2βe. When α<β, considering $L_{k}\geq 2$, we obtain that if $E_{i}^{k}\geq E_{j}^{k}$, then $\frac{\beta }{3}\leq \alpha <\beta$. Accordingly, if $\alpha <\frac{\beta }{3}$, $E_{i}^{k}$ might be smaller than $E_{j}^{k}$. Note that to guarantee a relatively high data correlation, $OR_{T}$ is often larger than 0.4 (as discussed in Section 5.3.2), and thus 0<α,β<0.6. If $\alpha <\frac{\beta }{3}<0.2$ ($OR_{i,k}>0.8$), then $E_{i}^{k}$ has some probability to be smaller than $E_{j}^{k}$. In our experiments, it is rare to achieve this strict condition in networks. Therefore, in general, $E_{i}^{k}>E_{j}^{k}$. The gathering node selection from the candidate parent node is more energy-efficient than from the neighbor node.

**Figure 4:**
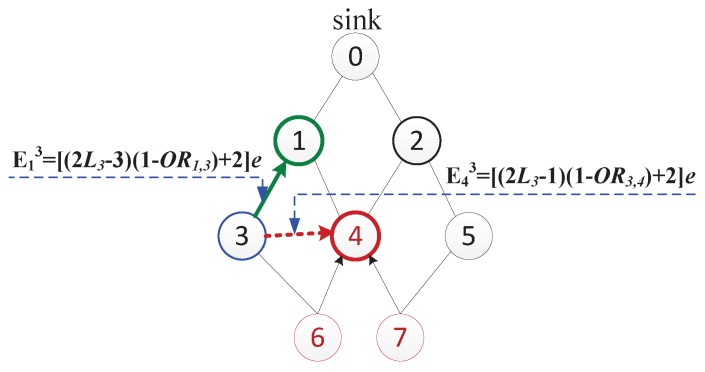
Gathering node selection for $v_{3}$.

For instance, we discuss about how $v_{3}$ (the current node) selects its gathering node from $v_{1}$ and $v_{4}$. In Figure 4, $L_{3}=2$, $OR_{1,3}=0.5$, $OR_{3,4}=0.7$. The total energy cost of transmitting data of $v_{3}$ to the sink via $v_{1}$ is $E_{1}^{3}=\left[\left(2L_{3}-3\right)\left(1-OR_{1,3}\right)+2\right]e=2.5e$, while that of selecting $v_{4}$ as the gathering node of $v_{3}$ is $E_{4}^{3}=\left[\left(2L_{3}-1\right)\left(1-OR_{3,4}\right)+2\right]e=2.9e$. Therefore, $E_{4}^{3}>E_{1}^{3}$, which is consistent with above analysis.

Case 2: $OR_{i,k}<OR_{T}$ and $OR_{j,k}\geq OR_{T}$. $v_{k}$ chooses $v_{j}$ as its gathering node, according to Rule 1. From Case 1 and Case 2, when $v_{k}$ gets $OR_{j,k}\geq OR_{T}$, $v_{k}$ selects its gathering node from the candidate parent nodes, and does not need to calculate the overlapping rates of sensing area with its neighbor nodes.

Case 3: $OR_{i,k}\geq OR_{T}$ and $OR_{j,k}<OR_{T}$. Node $v_{k}$ has two ways to send its data. One option is that $v_{k}$ becomes an independent node while $v_{j}$ is its relay node, and the total energy consumption of transmitting data of $v_{k}$ to the sink via $v_{j}$ is

(5) $$E_{j}^{k}=\left(2L_{k}-1\right)e.$$

The other option is taking $v_{i}$ as $v_{k}$’s gathering node, and the total energy cost is shown in Equation (3). Hence $E_{i}^{k}-E_{j}^{k}=\left[2-\left(2L_{k}-1\right)\left(1-\alpha \right)\right]e$. If $\left(2L_{k}-1\right)OR_{i,k}>2$, then $E_{i}^{k}<E_{j}^{k}$, and $v_{k}$ chooses $v_{i}$ as its gathering node. In one word, if there is no candidate parent node $v_{j}$ subject to $OR_{j,k}\geq OR_{T}$, a neighbor node $v_{i}$ with $OR_{i,k}\geq OR_{T}$ and $\left(2L_{k}-1\right)OR_{i,k}>2$ is selected as the gathering node of $v_{k}$. Furthermore, if no such neighbor node exists, $v_{k}$ becomes an independent node.

Case 4: $OR_{i,k}<OR_{T}$ and $OR_{j,k}<OR_{T}$. Considering the weak similarity, it is not necessary to aggregate the data from these nodes. Therefore, $v_{k}$ becomes an independent node, delivering its data to the sink without aggregation.

In conclusion, for free nodes, their candidate parent nodes have high priorities to be the gathering nodes. If all the candidate parent nodes have lower overlapping rates of sensing area than the threshold, then the neighbor nodes are considered to aggregate data.

**Rule 3.** *Data from every node is aggregated at most once, and in relay node selection, the non-gathering nodes take priorities over the candidate parent node with the most residual energy.*

Since data similarity is small between different gathering areas, data aggregation inter gathering areas probably has no significant advantages. Additionally, the data collection and processing for further aggregations may prolong the end-to-end delay. Consequently, in AggOR, taking into account the original data from each node, the aggregation executes at most once.

Obviously, the gathering nodes consume more power than the member nodes and the independent nodes, and thus are not suitable as relay nodes which need to contribute extra energy for data forwarding. Selecting non-gathering nodes as relays helps to balance the energy consumption in the entire network and prolong the network lifetime. If no non-gathering node exists, take the candidate parent node with the most remaining energy as the forwarder.

## 4. Implementation of AggOR Scheme

The transmission hierarchy diagram of WSN is constructed based on hello messages exchange between sensor nodes. Hello message includes the sender’s ID, coordinates, residual energy and the level. At the beginning, all sensor nodes initialize their levels as infinity, and the sink floods hello message including its level 0. Then other nodes update their levels after receiving hello messages. In specific, after receiving a hello message, $v_{i}$ checks if the difference of its stored level $L_{i}$ and the level in the hello message is larger than 1. If it is true, $v_{i}$ updates $L_{i}$ with the level in hello message plus 1, and then disseminates its hello message with the updated level; otherwise, if the level in the message is $L_{i}-1$, $v_{i}$ adds the ID in the message to its candidate parent node set $CP_{i}$; if the levels are the same, $v_{i}$ inserts the ID to its neighbor node set $N_{i}$.

**Figure 5:**
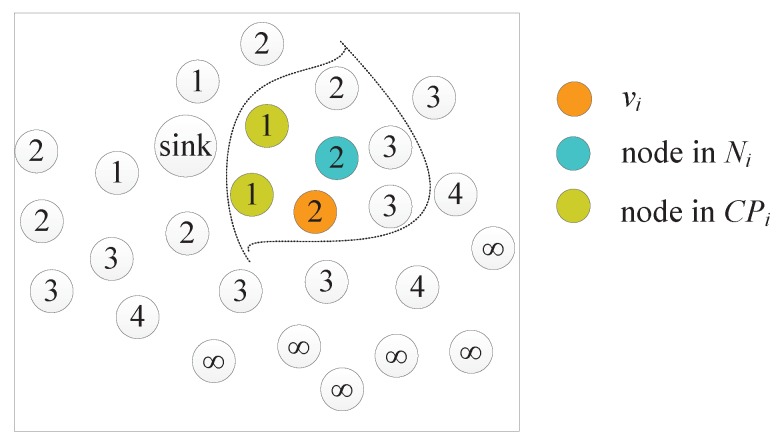
An instance topology under construction.

A topology under construction is illustrated in Figure 5 where the numbers are levels of nodes. Take node $v_{i}$ as an example; its $CP_{i}$ and $N_{i}$ are illustrated.

### 4.1. Gathering Area Construction

After transmission hierarchy diagram is completed, sensor nodes start to form the gathering area in a distributed manner. Gathering area construction begins from the lower-level free nodes to the upper-level layer by layer and follows the three aggregation rules in Section 3.2. In the construction process, a free node may be chosen as a gathering node in a new gathering area, or join in an existing gathering area as a member node, or become an independent node. Take $v_{i}$ as an instance; its gathering area construction algorithm is shown in Algorithm 1, a core of which is finding the candidate gathering node set as shown in Algorithm 2.


**Algorithm 1:** Gathering Area Construction.
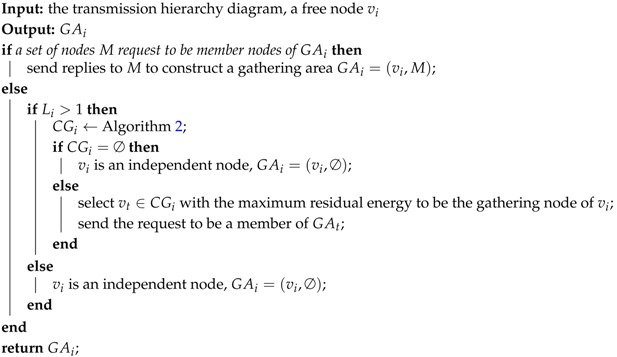



The network is initialized that all the sensor nodes are free nodes and there are (n+1) levels in the transmission hierarchy diagram. Levels are numbered as 0, 1, 2, …, *n*. Then the nodes at level *n* start constructing gathering areas firstly. For a node $v_{i}$ whose level is larger than 1, it calculates the overlapping rates of sensing area and obtains its candidate gathering node set $CG_{i}$ through Algorithm 2. If $CG_{i}$ is empty, $v_{i}$ becomes an independent node; otherwise, $v_{i}$ chooses the node $v_{t}$ in $CG_{i}$ which has the most residual energy as its gathering node, and sends the request to be a member node in the gathering area $GA_{t}$. For the free nodes at level 1, it is unnecessary to select the sink as gathering node, and their overlapping rates of sensing area with their neighbor nodes cannot be larger than 2 (Rule 2). Therefore each free node at level 1 turns into an independent node.


**Algorithm 2:** Finding Candidate Gathering Node Set.
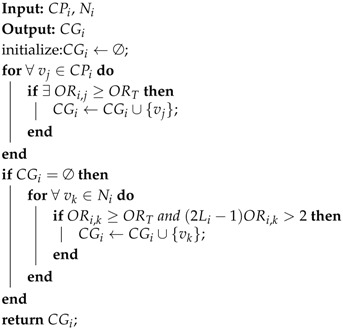



Algorithm 2 returns $CG_{i}$, as the set of candidate gathering nodes of $v_{i}$. For every candidate parent node $v_{j}$, if $OR_{i,j}\geq OR_{T}$, $v_{j}$ is included in $CG_{i}$. If there is no candidate gathering node selected from the candidate parent nodes, $v_{i}$ calculates the overlapping rates of sensing area with its neighbor nodes. If a neighbor node $v_{k}$ satisfies $OR_{i,k}\geq OR_{T}$ and $\left(2L_{i}-1\right)OR_{i,k}>2$, $v_{k}$ is included in $CG_{i}$.

### 4.2. Data Routing

In the process of data routing, member nodes send data to their gathering nodes through one hop transmission, while gathering nodes aggregate data collected in their gathering areas and then send them to the sink through the energy-efficient paths. Additionally, independent nodes send their own data to the sink without aggregation. The energy-efficient paths are established according to Rule 3, in which the non-gathering nodes are the first choice for relay nodes and then the candidate parent nodes with the most residual energy are selected as forwarders.

**Figure 6:**
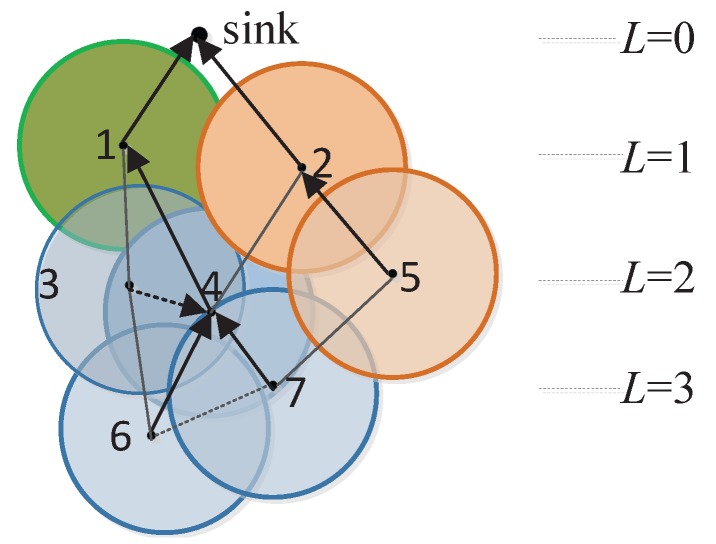
An example of AggOR implementation.

An instance of the construction of gathering areas and the data routing is depicted in Figure 6, which is the part in dotted circle of Figure 5. We suppose that $v_{4}$ has more residual energy than $v_{3}$, and $OR_{3,4}=0.7>OR_{T}$. All the nodes in the network are initialized as free nodes, i.e., $FN_{3}=\left\{v_{6},v_{7}\right\}$, $FN_{2}=\left\{v_{3},v_{4},v_{5}\right\}$ and $FN_{1}=\left\{v_{1},v_{2}\right\}$. At the beginning, $v_{6}$ and $v_{7}$, at the highest level 3, calculate the overlapping rates of sensing area with their candidate parent nodes. We get $OR_{4,6}>OR_{3,6}>OR_{T}$ and $OR_{4,7}>OR_{T}>OR_{5,7}$. Thus $CG_{6}=\left\{v_{3},v_{4}\right\}$ and $CG_{7}=\left\{v_{4}\right\}$. Due to more residual energy, $v_{4}$ is selected by $v_{6}$ and $v_{7}$ as their gathering node, $GA_{4}=\left(v_{4},\left\{v_{6},v_{7}\right\}\right)$. Then $FN_{3}=\emptyset$ and $FN_{2}=\left\{v_{3},v_{5}\right\}$. Next the free nodes $v_{3}$ and $v_{5}$ at level 2 construct their gathering areas. Similarly, regarding the candidate parent nodes, $OR_{1,3}<OR_{T}$, $OR_{2,3}<OR_{T}$ and $OR_{2,5}=OR_{T}$. Therefore, $CG_{3}=\emptyset$ and $CG_{5}=\left\{v_{2}\right\}$. Node $v_{5}$ chooses $v_{2}$ as it gathering node, $GA_{2}=\left(v_{2},\left\{v_{5}\right\}\right)$. Since $CG_{3}=\emptyset$,$v_{3}$ further calculates overlapping rate of sensing area with its neighbor node $v_{4}$, and gets $\left(2L_{3}-1\right)\times OR_{3,4}=3\times 0.7>2$. Because there is no other neighbor node, $CG_{3}=\left\{v_{4}\right\}$, and $v_{3}$ chooses $v_{4}$ as its gathering node, $GA_{4}=\left(v_{4},\left\{v_{3},v_{6},v_{7}\right\}\right)$. After that, $FN_{2}=\emptyset$ and $FN_{1}=\left\{v_{1}\right\}$. $v_{1}$ at the level 1 becomes an independent node, and $FN_{1}=\emptyset$. For the data transmission, $v_{3}$, $v_{6}$ and $v_{7}$ send their data to $v_{4}$, $v_{4}$ aggregates data in $GA_{4}$ and then chooses $v_{1}$ (non-gathering node) to relay its data to the sink. Node $v_{5}$ sends data to $v_{2}$ which aggregates and forwards data to the sink, while $v_{1}$ sends its data to the sink directly.

### 4.3. Complexity Analysis

In order to testify the validity and efficiency of AggOR, we analyze its complexity in terms of computation, message and storage complexity. The computation complexity of Algorithm 2 (finding candidate gathering node set) is $O(\max_{\forall i\in [1,m]}(|CP_{i}\cup N_{i}\left|)\right)$, where *m* is the number of sensor nodes in the network. It means in the worst case, a node $v_{i}$ visits all its candidate parent nodes $CP_{i}$ and its neighbor nodes $N_{i}$ to find its candidate gathering nodes $CG_{i}$. Accordingly the computation complexity of Algorithm 1 (gathering area construction) is $O(\max_{\forall i\in [1,m]}|CP_{i}\cup N_{i}|+|CG_{i}|)$. Since the number of elements in $CP_{i}$, $N_{i}$ and $CG_{i}$ are all less than *m*, the complexity of our algorithms is O(m).

In the process of transmission hierarchy diagram construction, sink starts flooding hello message and other nodes broadcast it after reception. Thus the control message cost is *m*. When constructing gathering areas, node $v_{i}$ sends join message to its potential gathering node, and after receiving acceptance message from it, $v_{i}$ sends acknowledgement message to join the gathering area. Hence in this process the message cost is 3m. Overall, the message complexity of AggOR is O(m).

For the control information, since each node stores its overlapping rates of sensing area with nearby nodes, candidate parent node set, neighbor node set, level and remaining energies of its candidate gathering nodes, the storage complexity is O(m). In addition, for the data packets, every member node only carries its own data, while each gathering node caches the data from its member nodes. Considering that data aggregation is implemented at one-hop distance, the number of data packets collected by a gathering node is usually far less than *m*, with the storage complexity O(m).

To sum up, compared with EEUC, whose message complexity is O(m), and HEER, whose message complexity is O(m) and computation complexity is exponential, our distributed scheme AggOR has relatively low complexities of computation, message and storage.

## 5. Performance Evaluation

### 5.1. Network Configurations

We evaluate the performance of AggOR scheme on OPNET Modeler [26] network simulation platform. The network configurations are listed in Table 2. Note that sensor nodes are evenly distributed in the monitoring field.

We select a typical scheme EEUC and a newly proposed scheme HEER to be our comparisons. EEUC is a distributed cluster mechanism where cluster heads are elected by localized competition. Through a function of the competition range Rcomp which is decided by the distance to the base station, several tentative cluster heads are elected to compete for final cluster heads. After the cluster head selection, other nodes join in their closest cluster heads. HEER, as a chain-based protocol, constructs clusters like LEACH, and establishes a Hamilton Path in each cluster to set an order for sensors to transmit data. In order to evaluate different gathering node selection methods, we take a variation of AggOR as comparison, in which the gathering nodes are only selected from candidate parent nodes (not considering the neighbor nodes), named AggOR-CP.

Note that the data sizes after aggregation are not the same in different schemes. In AggOR, after a gathering node aggregates *x* data packets, the amount of output data is p(0<p<x·d), which is decided by the overlapping rates of sensing area in this gathering area. Since the data aggregation function only removes duplicated data relevant with the overlapping sensing area, the whole data obtained by the sink are complete and accurate. However, in EEUC and HEER, a packet with a fixed amount of data is output by aggregating several data packets.

The following metrics are used for the performance evaluation.

(1) Network lifetime: the time interval from the beginning of the network to the death of the first node.
(2) Transmission overhead: the total amount of data transmitted in one data transmission round. It indicates the energy consumption of data sending and receiving in the whole network.
(3) Maximum number of hops to the sink: the maximum number of hops from sensor nodes to the sink in the network. More hops mean a longer time for which the sink has to wait to collect all the data in the scenario. Hence it implies the data delivery delay.
(4) Information accuracy: the ratio of the amount of information collected by the sink to the amount of information in all raw data.

Considering that sensor density may influence the performance of data aggregation, we will discuss about this issue in Section 5.3.1. In addition, the threshold of overlapping rate of sensing area is a significant factor affecting the size of gathering area and the energy efficiency of AggOR scheme. Therefore, we will analyze the influences of this threshold in Section 5.3.2. Moreover, we consider three scenarios corresponding to different shapes of monitoring field and different locations of the sink in the network. In Scenario SP, the nodes are deployed in a pyramid field, of which the top is the sink. In Scenario SC, the sink is placed at the center of a circular field. Scenario SS has a square field with the sink in the top-left corner. Note that in all the scenarios, sensor nodes are uniformly deployed. We will analyze the sources of gathering nodes in AggOR and AggOR-CP with different $OR_{T}$, in Section 5.3.3.

### 5.2. Experiment Results

**Figure 7:**
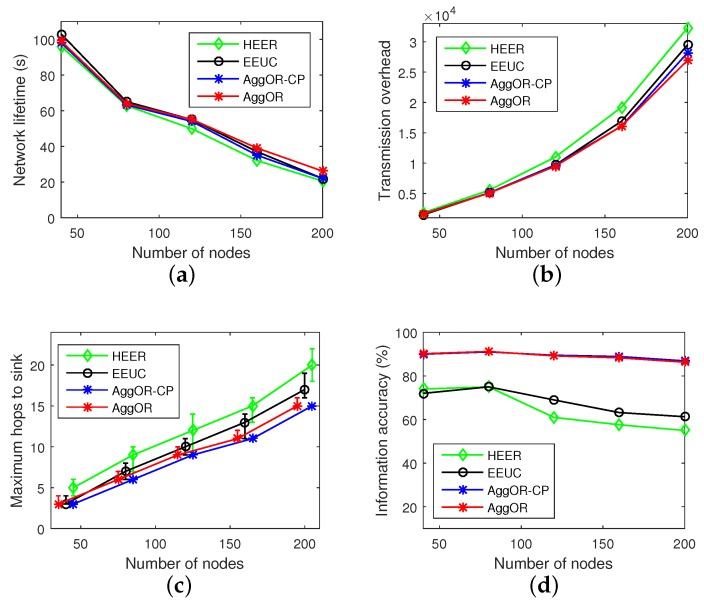
Experiment results. (**a**) network lifetime; (**b**) transmission overhead; (**c**) maximum hops to the sink; (**d**) information accuracy.

We evaluate the performances of four schemes, i.e., HEER, EEUC, AggOR-CP and AggOR, and the results are illustrated in Figure 7. Note that the experiments are conducted in Scenario SP. With the number of sensors increasing, the density of nodes does not change. In other words, the results are obtained under different network scales with the same density. Specifically, the networks with 40, 80, 120, 160 and 200 nodes cover approximately 20%, 40%, 60%, 80% and 100% of the whole 100×100 m${}^{2}$ scenario, respectively.

As Figure 7a shows, when there are 40 nodes in the network, EEUC has the longest lifetime of the network, and AggOR tightly follows. However, with the number of nodes increasing, the lifetime of AggOR which is longer than AggOR-CP, gradually exceeds EEUC from the scale of 120 nodes, and the lifetime of HEER is the shortest. Even though in EEUC scheme, the clusters output a single length-fixed packet after aggregation, which is smaller than the output of gathering nodes in AggOR, the main reasons of the result lie in two aspects. (1) The redundant data is forwarded for several hops in EEUC while redundancy is only relayed once in AggOR; (2) Compared with EEUC in which the cluster heads may be at lower levels, in AggOR, the gathering nodes are mainly the upper-level nodes, and thus sensor data are always forwarded up avoiding the back and forth relay.

**Figure 8:**
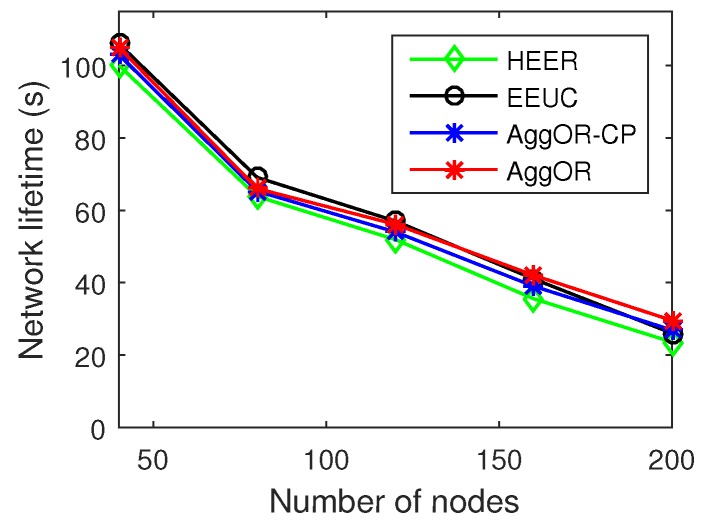
Another definition of network lifetime.

Note that there are different ways to define the dead time of the network. If the sensing range of the first dead node is covered by others, the network might continue to work. Therefore, beside the death of the first node, we analyze the network lifetime, which takes the time when some area cannot be sensed any longer or some data cannot be delivered to the sink as the dead time. The results are shown in Figure 8. The network lifetimes of all the four schemes in Figure 8 are a little longer than or the same as those in Figure 7a, because the sensing areas of some first dead nodes are covered by others and some first dead nodes are the gathering nodes or the forwarders for others. However, the trends of the results in these two figures are similar, both showing that our scheme AggOR achieves a similar network lifetime to EEUC and a little longer lifetime than HEER.

Figure 7b demonstrates that the transmission overhead in AggOR is a little smaller than those in AggOR-CP and EEUC schemes when the number of nodes is 80. As the number of nodes increases, the transmission overheads of HEER, EEUC and AggOR-CP are increasing faster than AggOR. AggOR has a smaller transmission overhead than AggOR-CP, EEUC and HEER by about 4%, 10% and 17% respectively in the scenario with 200 nodes. HEER always has the highest overheads among the compared schemes.

Because the maximum hops in multiple tests for HEER is not stable, we use median values rather than average values in Figure 7c. From the figure, AggOR-CP has the smallest and stable number of hops, while AggOR in some rare cases has more hops than others. This is because in our scenarios with $OR_{T}=0.5$, all the gathering nodes in AggOR-CP are the upper-level nodes, while there are some neighbor nodes as gathering nodes in AggOR, slightly increasing the number of hops in data transmission. As the number of nodes increases, the maximum hops of EEUC and HEER schemes are more unstable and larger than AggOR, especially HEER. When there are 200 nodes, the maximum hops in AggOR is smaller than EEUC and HEER by 12% and 25% respectively. The main reason is that member nodes send data to their gathering nodes by just one hop both in AggOR and AggOR-CP. By contrast, there exists multi-hop routing in clusters of EEUC, and in HEER the members transfer their data to cluster heads following Hamilton Paths, which increase hops to the sink and prolong the end-to-end delay.

As Figure 7d illustrates, in AggOR the ratio of information accuracy is the highest (around 88%) among those four schemes and very similar to AggOR-CP, which is higher than EEUC and HEER by 38% and 48% respectively. This is because aggregating lots of data into a small fixed amount of data and aggregating the same data for several times both affect the information accuracy. In HEER, the end node of Hamilton Path transmits its data to its neighbor which is closer to the cluster head, and the neighbor aggregates data into one packet of a constant size. This process continues until the data reaches the cluster head. Therefore, an original data may be aggregated for several times. In EEUC, the clusters aggregate all member data into one packet with fixed size, regardless of the specific redundancy ratios. By contrast, AggOR aggregates one data only once, and does not lose any information. Note that the ratio of information accuracy in AggOR is not 100% because of the redundant data.

In conclusion, AggOR scheme achieves an energy-efficient and quick data collection, while ensuring a high data accuracy. Moreover, AggOR has a greater advantage over other schemes when the network scale rises, because the efficiency of redundancy clearing within only one-hop forwarding is apparent in a large scale scenario.

### 5.3. Parameter Analysis

#### 5.3.1. Sensor Density Analysis

**Figure 9:**
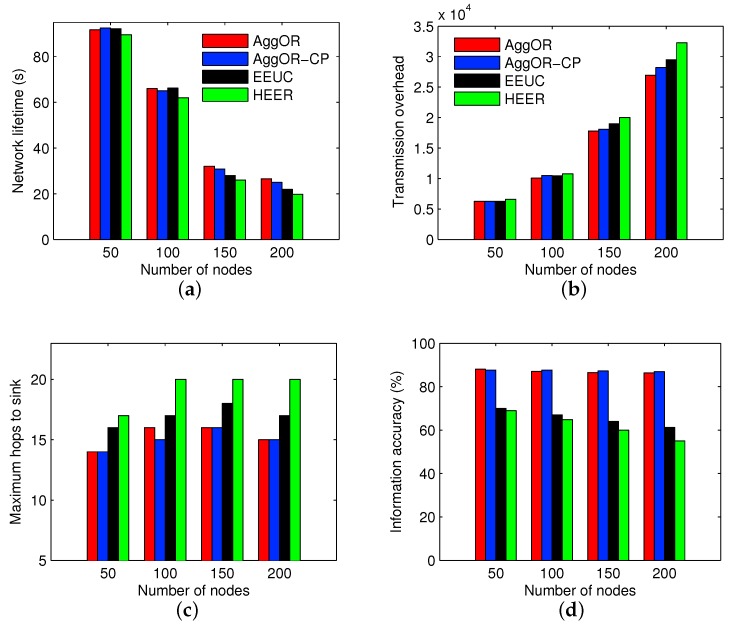
Results with different sensor densities. (**a**) network lifetime; (**b**) transmission overhead; (**c**) maximum hops to the sink; (**d**) information accuracy.

We analyze the effects of node density on the performances of all the four schemes in the fixed 100×100 m${}^{2}$ zone area. The numbers of nodes are 50, 100, 150 and 200, respectively. Accordingly, the sensor density ranges from 0.005 to 0.02 nodes per square meter. The results are illustrated in Figure 9.

In Figure 9, a high density leads to short network lifetimes and large transmission overheads for all the schemes, while the maximum hops to sink and the information accuracy do not change a lot. Additionally, as the sensor density increases, AggOR scheme, which always presents a high data accuracy and short transmission route in the experiments, begins to show longer lifetime and better energy efficiency than EEUC and HEER. On the whole, AggOR performs the best among the compared schemes with different sensor densities, especially in high-density sensor networks.

#### 5.3.2. Analysis of the Threshold of Overlapping Rate of Sensing Area

**Figure 10:**
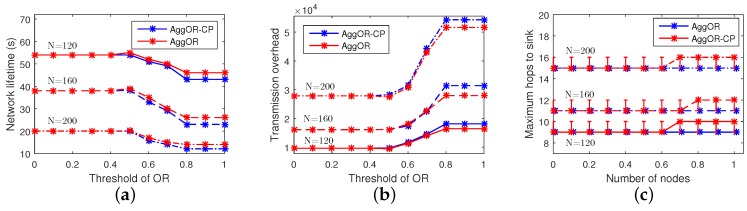
Results with different $OR_{T}$. (**a**) network lifetime; (**b**) transmission overhead; (**c**) maximum hops to the sink.

In order to show the impacts of the threshold of overlapping rate $OR_{T}$ on the overall performances of AggOR and AggOR-CP, we conduct a series of experiments in Scenario SP with $OR_{T}$ ranging from 0 to 1. Figure 10 shows the simulation results. The solid line, intermittent line and chain dotted line indicate the three scenarios with 120, 160 and 200 nodes, respectively. In Figure 10, the results are stable when $OR_{T}\leq 0.4$ or $OR_{T}\geq 0.8$, because in our dense WSN, all the overlapping rates of sensing area between two nodes are larger than 0.4 and smaller than 0.8.

In Figure 10a, when $OR_{T}\leq 0.4$, all gathering nodes are candidate parent nodes and almost no independent node exists. When $OR_{T}$ increases to 0.5, the network lifetime prolongs a little, because the gathering areas are constructed efficiently with the larger overlapping sensing area as well as some neighbor nodes selected as gathering nodes. When $OR_{T}$ continues to increase, less candidate parent nodes and more neighbor nodes are selected as the gathering nodes, which increases energy cost. Additionally, more independent nodes also limit the benefits of data aggregation. Therefore, with a medium value 0.5 of $OR_{T}$, AggOR reaches the longest network lifetime. Moreover, the network lifetime of AggOR is superior to that of AggOR-CP.

Figure 10b depicts that, when there are 120 or 200 nodes, the smallest transmission overhead appears in AggOR when $OR_{T}$ is 0.5. It is obvious that the larger $OR_{T}$ is, the higher transmission overhead there is due to less aggregation. In addition, transmission overhead in AggOR is smaller than AggOR-CP, and large scale leads to a bigger difference between them. It is consistent with our previous analysis that the neighbor nodes working as gathering nodes cost less energy for data transmission than independent nodes.

Data collection and aggregation by the gathering nodes cause a little delay apparently, and the neighbor nodes as the gathering nodes further increase the hops to the sink. When $OR_{T}$ rises, the median value of maximum hops to the sink in AggOR is bigger than AggOR-CP, as shown in Figure 10c. However, the largest difference between them appears when $OR_{T}$ is larger than 0.6 and the difference is one hop, which implies that there are no candidate parent nodes suitable to be gathering nodes for some node on the longest path to the sink.

#### 5.3.3. Gathering Node Analysis

**Figure 11:**
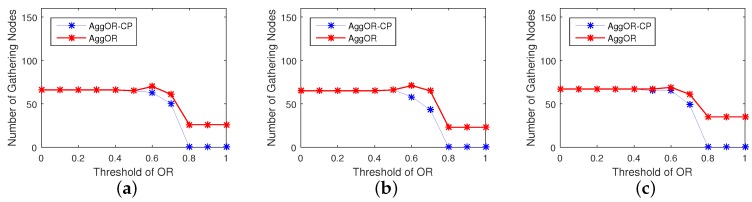
The numbers of gathering nodes in three scenarios. (**a**) Scenario SP; (**b**) Scenario SC; (**c**) Scenario SS.

**Figure 12:**
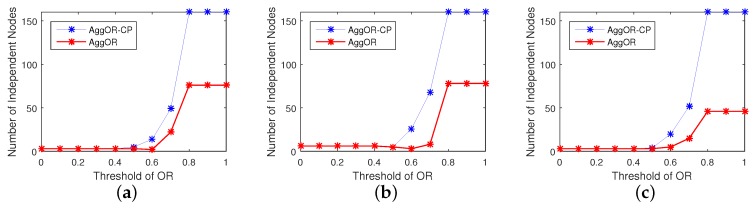
The numbers of independent nodes in three scenarios. (**a**) Scenario SP; (**b**) Scenario SC; (**c**) Scenario SS.

Considering that the number of gathering nodes indicates the efficiency of data aggregation, we analyze the number of gathering nodes and the number of independent nodes, in three scenarios, i.e., SP, SC and SS, with 160 nodes. The results are illustrated in Figure 11 and Figure 12.

In different scenarios, the numbers of two kinds of nodes are similar with the same $OR_{T}$. Comparing the numbers of gathering nodes in AggOR and AggOR-CP in Figure 11, we get that when $OR_{T}$ is smaller than 0.5, no neighbor nodes are selected as gathering nodes. In Figure 12, when $OR_{T}$ increases from 0.5, AggOR-CP has more independent nodes than AggOR. When $OR_{T}$ is larger than 0.8, the numbers of gathering nodes and the numbers of independent nodes both reach plateaus. Specifically, all nodes in AggOR-CP are independent nodes, while AggOR has some neighbor nodes selected as gathering nodes, and hence reduces the transmission overhead.

**Figure 13:**
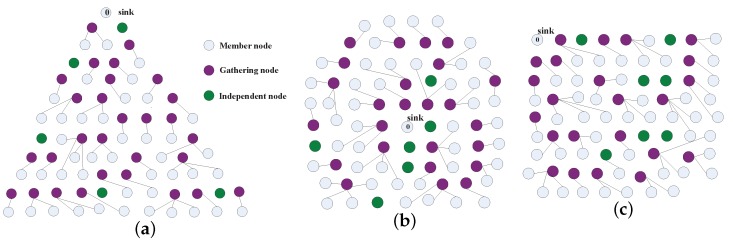
Sketch maps of three scenarios. (**a**) Scenario SP; (**b**) Scenario SC; (**c**) Scenario SS.

Figure 13 shows the sketch maps of three scenarios, i.e., SP, SC and SS, with 80 nodes in AggOR. The edges indicate the relationships between gathering nodes and member nodes. Due to the limited spaces, to avoid confusion caused by crossing lines, we do not show the data transmission paths from the gathering nodes to the sink. From the figure, we get that the gathering nodes are usually located near their member nodes, and most of them are closer to the sink than member nodes.

Overall, the larger the sensor density is, the better AggOR performs. In a relatively dense network, a larger network scale leads to a greater advantage of AggOR over EEUC and HEER. Additionally, $OR_{T}$ has a significant effect on the performance of AggOR, which peaks at $OR_{T}=0.5$ in our experiments. In real scenarios, an appropriate $OR_{T}$ can be obtained through sampling analysis during preliminary study. Furthermore, AggOR has a longer network lifetime and a smaller traffic overhead than AggOR-CP in the three scenarios with different sensor deployments and different locations of the sink.

## 6. Conclusions

Wireless sensor networks are deployed to support a variety of precise monitoring applications in smart factories, and require energy-efficient and no-entropy-loss data aggregation. In this paper, we propose a data aggregation scheme based on the overlapping rate of sensing area, named AggOR. In the transmission hierarchy diagram, some candidate parent nodes as well as appropriate neighbor nodes, whose overlapping rates of sensing area are not smaller than a preset threshold $OR_{T}$, may be selected as the candidate gathering nodes. It guarantees that the sensor data in a gathering area are extremely correlative, and there exist a large amount of redundant data to be cleaned. Member nodes transfer their data to gathering nodes through one hop, and only duplicated data are removed by aggregation, ensuring a short end-to-end delay and a high data accuracy. A large quantity of experiments on OPNET modeler show that AggOR has a better data accuracy and a shorter delay than compared schemes, while keeping similar network lifetime.

However, the specific relation between $OR_{T}$ and network density still requires further study. In addition, considering the occurrence of multiple events at the same time [27], how to optimize multi-event data collection by analyzing overlapping sensing area is another research topic for the future.

## Figures and Tables

**Table 1:** Symbols.

Symbol	Description
$R_{S}$	Sensing radius of a sensor node.
$R_{C}$	Communication radius of a sensor node.
$E_{T}$	Consumed energy of sending per unit data.
$E_{R}$	Consumed energy of receiving per unit data.
*d*	The size of data collected by a sensor node.
$OR_{i,j}$	Overlapping rate of sensing area of two nodes $v_{i}$ and $v_{j}$.
$OR_{T}$	Threshold of overlapping rate of sensing area.
$GA_{i}$	Gathering area with $v_{i}$ as the gathering node.
GN	Gathering node, which aggregates the data collected in a gathering area.
$CG_{i}$	Candidate gathering node set of $v_{i}$.
$CP_{i}$	Candidate parent node set of $v_{i}$, including all the upper-level nodes that couldcommunicate with $v_{i}$ directly.
$N_{i}$	Neighbor node set of $v_{i}$, which consists of the nodes at the same level that couldcommunicate with $v_{i}$ directly.
$L_{i}$	Level of $v_{i}$, and $L_{i}\in N^{+}$.
$d_{i}^{T}$	The total amount of data transferred from $v_{i}$.
$d_{i}^{R}$	The total amount of data received by $v_{i}$.
$E_{GN_{i}}$	The energy consumed for delivering aggregated data from the gathering node $v_{i}$.
$E_{GA_{i}}$	The total energy cost of the nodes in the gathering area $GA_{i}$.
$E_{i}^{k}$	The total energy cost of transmitting data of node $v_{k}$ to the sink via another node $v_{i}$.
$FN_{n}$	Free nodes at the level *n*.

**Table 2:** Simulation parameters.

Parameter	Value
Scenario (m${}^{2}$)	100×100
Number of sink node	1
Number of sensor nodes, *N*	40, 80, 120, 160 and 200
Sensing radius (m)	25
Communication radius (m)	52
Data collection cycle (s)	60
$OR_{T}$	0.5
